# Severe neurological crisis in adult patients with Tyrosinemia type 1

**DOI:** 10.1002/acn3.51160

**Published:** 2020-08-21

**Authors:** Charlotte Dawson, Radha Ramachandran, Samreen Safdar, Elaine Murphy, Orlando Swayne, Jonathan Katz, Philip N. Newsome, Tarekegn Geberhiwot

**Affiliations:** ^1^ Department of Diabetes, Endocrinology and Metabolism Queen Elizabeth Hospital Birmingham NHS Foundation Trust Birmingham United Kingdom; ^2^ Adult Inherited Metabolic Disease Unit Guy’s and St Thomas NHS Foundation Trust London United Kingdom; ^3^ Charles Dent Metabolic Unit National Hospital for Neurology and Neurosurgery London United Kingdom; ^4^ Neurorehabilitation Unit National Hospital for Neurology and Neurosurgery London United Kingdom; ^5^ Department of Endocrinology Royal Free London NHS Foundation Trust London United Kingdom; ^6^ National Institute for Health Research Birmingham Biomedical Research Centre at University Hospitals Birmingham NHS Foundation Trust Birmingham United Kingdom; ^7^ Centre for Liver & Gastrointestinal Research Institute of Immunology and Immunotherapy University of Birmingham Birmingham United Kingdom; ^8^ Institute of Metabolism and Systems Research University of Birmingham Birmingham United Kingdom

## Abstract

We report six adult patients with Tyrosinaemia type 1 (HT‐1) who presented with recurrent porphyria‐like neurological crises after discontinuation/interruption of 2‐(2‐nitro‐4‐trifluoro‐methylbenzyol)‐1,3 cyclohexanedione (NTBC) treatment. The crises were life‐threatening for some of the patients, with respiratory muscle paralysis requiring ventilatory support, hemodynamic disturbance due to autonomic changes requiring resuscitation, acute progressive ascending motor neuropathy causing profound impairment, recurrent seizures, and neuropathic pain. Our patients’ porphyria‐like presentations were variably misdiagnosed, with delay to diagnosis resulting in more severe recurrent attacks. We report the first series of neurological crises in adult patients with HT‐1. These crises, which may be fatal, can be prevented and treated effectively with neurologist/physician awareness and patient education.

## Introduction

**Figure 1:**
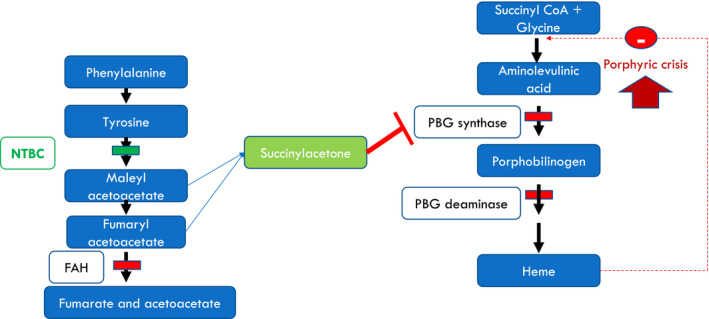
Metabolic pathways and the effect of NTBC. Deficiency of fumarylacetacetate hydrolase enzyme leads to accumulation of the metabolites upstream. An alternative pathway then leads to accumulation of succinylacetone. Succinylacetone inhibits the second step of heme biosynthesis leading to accumulation of aminolevulinic acid and hence a clinical picture resembling that of acute intermittent porphyria. NTBC prevents the accumulation of the toxic metabolites.

Tyrosinemia type 1(HT‐1) is a rare autosomal recessive inherited metabolic disorder (OMIM #276700) caused by a defect in fumarylacetoacetate hydrolase (EC: 3.7.1.2), a key enzyme in the final step of tyrosine metabolism. As a result of the metabolic block, toxic metabolites such as succinylacetone, maleylacetoacetate, and fumarylacetoacetate accumulate in tissues and body fluids (Fig. 1).^1^ HT‐1 usually manifests in the first year of life with failure to thrive, acute and chronic liver failure, a Fanconi‐like syndrome, renal failure, neurological crisis resembling acute intermittent porphyria (AIP), and hepatocellular carcinoma. If untreated, for most patients, life expectancy rarely extends beyond 3 years.^2^ Since the introduction of 2‐(2‐nitro‐4‐trifluoro‐methylbenzyol)‐1,3 cyclohexanedione (NTBC) as a treatment in 1992,^3^ the natural history of the disease has transformed, with almost all early treated patients now surviving to adulthood. This medical success story relies on parents’ and patients’ perseverance in adhering to treatment which includes lifelong medication and natural dietary protein restriction, in conjunction with tyrosine‐ and phenylalanine‐free protein substitutes.

The clinical consequences of NTBC discontinuation on subsequent neurological crises are not well‐documented and there is no published evidence to guide how best to care for these patients during such crises. To our knowledge, this is the first published description of adult HT‐1 patients’ pre‐ or post‐NTBC therapy.

## Case Reports

We report the United Kingdom experience of six patients with HT‐1 from three adult metabolic centers who presented with a porphyria‐like neurological crisis after discontinuing or interrupting their NTBC treatment.

The patients’ characteristics and clinical course are shown in Table 1. The majority were diagnosed in infancy following an initial presentation with liver failure and were commenced on NTBC shortly afterwards. Hepatic and renal complications resolved on treatment and all patients were well throughout childhood and adolescence with no further hepatic and/or renal complications. The mean ± SD age at the time of the most severe neurological crisis in adulthood was 23 ± 3 years. The patients experienced between 1 and 15 episodes each of varying severity, with a cumulative total of at least 40 hospital attendances. All patients had variable or intermittent compliance with their NTBC treatment during the months leading up to their acute neurological presentation. Although some patients were aware of the risk of a neurological crisis if they did not take their medication, they principally thought of their condition as a “liver disease.” Consequently, when they presented to the emergency department (ED) the connection between nonadherence to NTBC treatment and neurological decompensation was often not made and therefore did not form part of the initial differential diagnosis.

**Table 1:** Patient characteristics, clinical presentation, and outcome.

Patient (M/F)	Initial presentation	Age at diagnosis (months)	Age at start of NTBC (months)	Age at time of most severe crisis (years)	Period of NTBC dis‐ continuation (days)	Number of crises requiring A&E attendance	Presentation during severe crisis	Initial diagnosis given during A&E attendance	Outcome
1, M	Raised tyrosine on newborn screening	0.3	3	26	Intermittent compliance since childhood	1	Confusion, abdominal pain, peripheral neuropathy/global motor weakness	Guillain‐Barré syndrome/porphyria	Prolonged ventilatory support – 6 weeks
									Prolonged neuromuscular recovery (8 months hospitalization, 11 months to full recovery)
2, M	E‐coli sepsis/Liver failure	1	5	21	Intermittently over 6 months	4, over 6 weeks	Confusion, seizure	Viral encephalitis/UTI	Ventilatory support required
									Full recovery within 2 weeks
3, M	Liver failure	2	3	25	Intermittently over several years	15, over 7 years	Painful extremities, abdominal pain, blurred vision, and seizure	Porphyria/viral illness	Full recovery with each episode within 1–2 weeks
4, F	Liver failure	8	8	22	Intermittently over 5 weeks	7, over 5 years	Abdominal pain, intractable vomiting	Small bowel obstruction	Full recovery with each episode within 2–4 days
5, F	Liver failure	1.2	6	18	Intermittently over 12 years	12, over 8 years	Confusion, seizure.	Pseudo‐seizures/intractable pain with no organic cause	Prolonged ITU stay with ventilator support required
6, M	Liver dysfunction	1.2	1.2	24	Variable compliance	2 severe and multiple episodes of less severe symptoms over 7 years	Abdominal pain, painful extremities, vomiting	Tyrosinemia related acute porphyric crisis	Full recovery within 5 days

### Case descriptions of each patient’s most clinically severe episode

#### Case 1

A 26‐year‐old man, known to be intermittently adherent to prescribed NTBC medication, presented with confusion, agitation, and hallucinations whilst on a trip abroad. He developed severe abdominal pain, anorexia, and vomiting. He was transferred back to a UK hospital 4 days after onset of these initial symptoms. Over the course of a few days he developed global motor weakness, peripheral neuropathy, and respiratory failure requiring intubation. The initial differential diagnosis included Guillain‐Barre Syndrome. A 6 month ITU admission was followed by a 4 month period of intensive inpatient rehabilitation. He had residual lower limb weakness on discharge, which resolved by 1 year following presentation.

#### Case 2

A 21‐year‐old man, who had only intermittently taken his prescribed NTBC over the previous 6 months, was brought to the ED following two generalized tonic‐clonic seizures. On admission, he was in respiratory distress, agitated and combative with a severe bite mark on his tongue. He required intubation and ventilation in intensive care. Although hypertensive he was clinically hypovolemic and hyponatremic (Na 127 mmol/L). He had mild neutrophilia with normal C‐reactive protein (CRP). Infective encephalitis was considered in the differential. Treatment included antiviral, antifungal and antibiotic medications with cautious fluid replacement.

#### Case 3

A 25 year old man presented to the ED with irritability, painful extremities, severe abdominal pain, blurred vision and a generalized tonic‐clonic seizure after he discontinued his NTBC treatment 1 month prior to this. On admission he was hypertensive and tachycardic. He was treated with supportive measures, heme arginate infusion and NTBC and symptoms resolved.

#### Case 4

A 22 year old female presented to the ED with a one week history of severe lower abdominal pain, intractable vomiting and constipation after stopping NTBC for 2 weeks. Despite documented hypertension (147/98), clinically she was hypovolemic, and was admitted under the surgical team after fluid resuscitation. An abdominal X‐ray showed fecal impaction with dilated bowel but a CT abdomen did not reveal any bowel obstruction. She had no evidence of infection with a normal white cell count, CRP, and serum amylase. Her symptoms improved rapidly upon resumption of NTBC and supportive therapy.

#### Case 5

A 26 year old female was brought to the ED with confusion following a generalized tonic‐clonic seizure. A week prior to her admission she suffered from severe abdominal pain and vomiting after stopping NTBC. During admission, she had persistent tachycardia, hyponatremia (Na 110 mmol/L), and developed severe proximal muscle weakness with type 2 respiratory failure, requiring intubation and later tracheostomy for slow respiratory weaning. Neurophysiological studies showed proximal patchy neuropathy and myopathy. She recovered upon resumption of NTBC and after extensive rehabilitation.

#### Case 6

A 24 year old male presented to the ED with a 3 day history of worsening abdominal pain, vomiting and poor oral intake. During admission he had tachycardia (120–130 beats per minute) and severe pain in his abdomen and extremities. He required nasogastric nutrition. Management included doubling of his usual dose of NTBC, tyrosine‐free amino acid supplements, hydration (both via enteral feed and intravenously), and propranolol to control tachycardia.

Table 2 summarizes the clinical and biochemical characteristics of the most severe episodes. As with acute intermittent porphyria crises, abdominal pain, painful extremities, hypertension, and hyponatremia (Na = 110–127 *μ*mol/L) were common findings.

**Table 2:** Clinical and biochemical features during acute crises.

Findings	Patient 1	Patient 2	Patient 3	Patient 4	Patient 5	Patient 6
Confusion	Yes	Yes	Yes	No	Yes	No
Seizures	No	Yes	Yes	No	Yes	No
Muscle weakness	Yes	Yes	No	No	Yes	No
Pain in extremities	Yes	Yes	Yes	Yes	Yes	Yes
Abdominal pain	Yes	Yes	Yes	Yes	Yes	Yes
Vomiting	Yes	Yes	Yes	Yes	Yes	Yes
Hypertension (SBP> 140)	Yes	Yes	Yes	Yes	Yes	No
Hyponatraemia (Na < 130 mmol/L)	No	Yes	Yes	Yes	Yes	No
ALT> 1.5 ULN	ND	No	No	No	No	No
INR> 1.2	ND	Yes	No	No	No	No
Increased urine porphyrin/creatinine ratio (>40)	Yes	—	Yes	y	—	—
Increased urine ALA/creatinine (>10 *μ*mol/mmol)	Yes	—	—	Yes	—	—
Increased blood spot Succinylacetone	Yes	Yes	Yes	Yes	Yes	Yes
NTBC level below therapeutic range	Yes	Yes	Yes	Yes	Yes	No
Others	Acute kidney injury ‐ probably related to gentamicin – resolved on stopping antibiotic					

ND, not done.

In all, severe neurological crises were managed by supportive therapy and NTBC (re)commencement. All patients had an eventual full recovery within a time interval ranging from 2 weeks to 12 months.

## Discussion

This case series illustrates the severity, relatively common occurrence, and challenge of acute neurological crisis management in young adults with HT‐1. It emphasizes the need for vigilance and education of both clinicians and young adults with HT‐1 about the risk of this life‐threatening but preventable neurological crisis. As there are no recommendations on the management of porphyria‐like crises in HT‐1, based on our experience and pre‐NTBC evidence, we sought to review crisis management.

In the largest cohort published to date, 104 hospital admissions due to neurological crisis were reported in 20 of 48 children with HT‐1 during the first year of life prior to treatment with NTBC.^4^ Untreated childhood HT‐1 is therefore well‐known to be associated with neurological crisis and treatment with NTBC has effectively eliminated this risk. However, when adolescent or adult patients discontinue or only very intermittently take NTBC, there is also a high likelihood of developing a neurological crisis. This is the first case series of adults with HT‐1 presenting with recurrent crises, although a single adolescent case has previously been reported.^5^ Typically, the crises followed a course of poor treatment compliance associated with recurrent abdominal pain and vomiting. During these severe crises patients presented with seizures, confusion, painful paraesthesia, autonomic signs, hyponatremia, self‐mutilation, respiratory muscle paralysis and progressive ascending paralysis. These neurological crises in adults had a similar presentation and course as those reported in three children whose treatment with NTBC was interrupted.^6^, ^7^, ^8^

Succinylacetone, the abnormal metabolite which accumulates in patients with HT‐1, is an extremely potent competitive inhibitor of *δ*‐aminolevulinic acid (ALA) in the heme biosynthesis pathway (Fig. 1).^9^ Due to the biochemical and clinical resemblance of HT‐1 associated neurological crises and acute intermittent porphyria (AIP), this group of symptoms in HT‐1 patients is sometimes referred to as a porphyria‐like syndrome. The only precipitant that is required to cause this type of acute crisis is interruption to NTBC therapy. None of the patients described here reported another metabolic stressor (e.g. infection or fever) triggering their crisis.

Our patients’ presentations were variously misdiagnosed as urinary tract or CNS infections, acute abdomen, epilepsy, or Guillain‐Barré syndrome. In addition, some patients were labeled as having a “functional disorder” which resulted in mistrust of care providers, more severe subsequent recurrent attacks and disability. This indicates a lack of awareness of the possible re‐emergence of HT‐1 neurological manifestations not only by the admitting local clinical team but also, occasionally, by specialist care providers. Patients may not always make the association between not taking their NTBC medication and a neurological crisis, and a medical team, unfamiliar with the condition, may simply record a list of prescribed medications without closely enquiring how often they are taken.

The approach for treating crises in our patients was: (1) airway protection and respiratory support during paralysis, prompt treatment of fluid and electrolyte imbalance, and symptom control for seizures, pain, vomiting, and tachycardia as per acute porphyria emergency management national guidelines (www.BIMDG.org.uk); (2) disease modifying therapy by the provision of adequate calorie intake and the immediate urgent reintroduction of NTBC orally or via NG tube; and (3) return to their usual phenylalanine and tyrosine restricted diet within 24 to 48 h. One patient also received heme arginate. Intravenous glucose decreases endogenous protein breakdown, as well as inhibiting the porphyrin‐synthesizing enzyme δ‐aminolevulinic acid synthase and hence plays a dual protective role. The use of heme arginate to treat acute porphyric‐like crises associated with HT‐1 has only been reported in very few cases and its potential benefit has not been formally evaluated.^5^, ^10^ Respiratory failure and seizures can develop rapidly, and so regular clinical monitoring of neurological and respiratory status is important.

In conclusion, when patients with HT‐1 seek medical help with nonspecific symptoms such as pain, vomiting, seizure, and/or weakness, a porphyria‐like neurologic crisis should be considered in the differential and managed promptly.

## Conflict of Interest

All authors declare that they do not have a conflict of interest.
